# Dr. Cayley, on Opium in Costiveness

**Published:** 1806-04

**Authors:** G. Cayley

**Affiliations:** Ripon


### *To the Editors of the Medical and Physical Journal.*

GENTLEMEN,

HAVING read in the 84th Number of your Journal, the history of a remarkable case of costiveness in a young woman, treated by Mr. Earnest, of the Sheffield Infirmary, which did not yield to the combined powers of the most active purgatives, but at length, medicines being laid laid aside, a cure was effected by the secret operations of nature. Upon this case, Mr. Earnest makes the following just observation, of which he will most likely not lose sight in his future practice in similar cases; and if what I have to add, should be deemed worthy of a place in your Journal, and be acceptable to Mr. Earnest and the rest of your Readers, I shall be gratified.

My remarks, gentlemen, are not intended as any thing very novel or extraordinary in the practice of medicine, but as a monitory exemplification to many who are too much in the habit of relying solely on strong cathartics, without reflecting that, in many instances, the application of such remedies, if continued for a length of time, will by their astringency increase the disorder, which it is their object to remove. As I have been particularly fortunate in the treatment of a great many bad cases of a similar description, I shall subjoin some of the most remarkable ones; the modus medendi will confirm the opinion which Mr. E. has offered, as to what might have been the result of a contrary practice. He says, " perhaps, had antispasmodics and cathartics been conjoined, along with the use of the warm bath, their general remedial powers might have proved more effectual and beneficial."

The first case I shall relate is that of a gardener, aged 28, of middling stature, stout, and had enjoyed good health, until six months previous to his consulting me, which was for the following most distressing disorder. His head was afflicted with the most violent pains; he was never free from pains and spasms in the muscles of the hind head and neck, his penis was contracted in a most astonishing manner; it was with the utmost difficulty that he could make water, and never had above one stool in fifteen or sixteen days; his sleep was much disturbed, his appetite impaired, and spirits much depressed; he told me that he had taken purgatives of every kind, without having experienced the least relief from them, and had become miserable under his sufferings.

I began my treatment by ordering him one grain of opium and one of digitalis purpurea every three hours; this very soon had the desired effect, the bowels and urinary organs began to perform their functions, and he continued those sovereign remedies for a considerable length of time; suspending them occasionally, and interposing small doses of calomel, his cure was completed by tonics.

Case 2d.—A lady, rather advanced in life, had a violent bowel complaint with costiveness, not yielding to the most most powerful cathartics; had incessant vomiting, and threw up many gall stones. I was called in at this critical time, and ordered her opii. gr. ij. calomelan. gr. x. to be taken immediately, and to be put into a warm bath; in a short time she had a gentle motion; in a few hours I was under the necessity of giving her a solution of the natron vitriolat. which produced two more evacuations, but in three days she died.

Cases 3d. and 4th.—Two female patients, both advanced in life, had the most alarming disorders in their bowels, their stomachs rejecting every thing, and the injections which were made of a decoction of chamomile flowers, oil, and magnesia vitriolata, were strongly tasted in their mouths; to both of them I gave the same remedies as in Case, No. 2. They both of them were speedily relieved, and were very soon completely restored to their former healthy state; the three last patients had been bled before I was called in.

Case 5. A very corpulent woman, who had had several children, had at this present time a most difficult labour, of five days, attended with the most painful and distressing circumstances; she had been delivered only two days and a half when I was called in, and had not had a passage for eight days, though she had been bled, and had taken strong purgatives. The gentlemen who attended her, did not apprehend that there had been any inflammation; one of them was pretty confident there had not; I was of a contrary opinion. I found the woman evidently dying, but was desirous of procuring a passage, which injections would not effect. I gave her opii. gr. j. calomel. gr. vi. Pil. e colocynth. comp. gr. x. In four hours she had two gentle motions, after which she took tinct. opii. gtt. xx. in a cordial draught, and had wine and water given her, with gruel and broth; in the evening she had another good motion, but died early in the morning. I am of opinion, that an earlier application of the above remedies might have proved more beneficial.

Case 6. This shall be the last case I shall trouble you with, though I could enumerate others equally as important.

A farmer, about forty years of age, sent for me on the 9th day of an obstinate bowel complaint; nothing could pass him; his stomach rejected every thing, and he was in the most violent pain; his apothecary had been with him in the morning, and assured him he could do nothing more for him.

him. As he lived at some distance from hence, and at an equal distance from the apothecary, and being late at night when I was sent for, and the messenger telling me, as correctly as he could, the nature of the man's case; I took two papers of calomel, one of eight grs. and the other of five grs. and a small quantity of laudanum. Upon my arrival at this good man's house, I found him in great pain; his abdomen was excessively hard; his stomach rejected most things; his pulse did not indicate inflammation: the purgatives and injections, that had been administered, proved ineffectual. I ordered the belly to be fomented, and gave him the eight grs. of calomel, and forty drops of laudanum; in one hour he found himself much easier; in two hours thought he should have a stool. I then left him, ordering, that if he had not a stool in one hour more, to give him the five grs. of calomel, and xx gtts. tinct. opii. After the second stool, I wrote to the apothecary, requesting he would see him in the morning, and if he had no evacuations, to give him the natron vitriolat. with senna; and if that did not succeed, and the pains return, that he should take some blood from him, and apply a blister to the abdomen; the former part of my plan did produce some effect, but not to that extent that the apothecary thought was the desideratum, consequently he pursued the whole of my plan, with the happiest effect.

This is all that I have to state with respect to Mr. Earnest's paper, and have only to observe, that the treatment I have here laid down, requires the greatest caution, and ought not to be attempted indiscriminately by the young beginner. I am, &c.

